# Participation in the Cardiovascular Health Awareness Program (CHAP) by older adults residing in social housing in Quebec: Social network analysis

**DOI:** 10.1186/s12913-020-06019-2

**Published:** 2021-01-07

**Authors:** Nadia Deville-Stoetzel, Janusz Kaczorowski, Gina Agarwal, Marie-Thérèse Lussier, Magali Girard

**Affiliations:** 1 Department of Sociology, University of Québec in Montreal (UQAM), Montreal, Canada; 2 Department of Family and Emergency Medicine, University of Montreal, and University of Montreal Hospital Research Centre (CRCHUM), Montreal, Canada; 3 Department of Family Medicine, McMaster University, Hamilton, Canada; 4 Department of Health, Aging and Society, McMaster University, Hamilton, Canada; 5 Department of Health Research Methods, Evidence and Impact, McMaster University, Hamilton, Canada; 6 University of Montreal, Montreal, Canada

**Keywords:** Community-based participatory research, Cardiovascular diseases, Sociometric technics

## Abstract

**Background:**

The Cardiovascular Health Awareness Program (CHAP) is as a community-based cardiovascular disease prevention program recently adapted to target older adults living in 14 social housing buildings in Ontario (7) and Quebec (7). Social network analysis (SNA) has been used successfully to assess and strengthen participation in health promotion programs. We applied SNA methods to investigate whether interpersonal relationships among residents within buildings influenced their participation in CHAP.

**Methods:**

Our aim was to examine relational dynamics in two social housing buildings in Quebec with low and high CHAP attendance rates, respectively. We used sociometric questionnaires and network analysis for the quantitative phase of the study, supplemented by a phase of qualitative interviews. All residents of both buildings were eligible for the sociometric questionnaire. Respondents for the qualitative interviews were purposively selected to represent the different attendance situations following the principle of content saturation.

**Results:**

In total, 69 residents participated in the study, 37 through sociometric questionnaires and 32 in qualitative interviews. Of the latter, 10 attended almost all CHAP sessions, 10 attended once, and 12 attended none. Results of the quantitative analysis phase identified well-known and appreciated local leaders. In Building 1, which had a high attendance rate (34.3%), there was a main leader (in-degree or ‘named by others’ frequency 23.2%) who had attended all CHAP sessions. In Building 2, which had a low attendance rate (23.9%), none of the leaders had attended CHAP sessions. Results of the qualitative analysis phase showed that residents who did not attend CHAP sessions (or other activities in the building) generally preferred to avoid conflicts, vindictiveness, and gossip and did not want to get involved in clans and politics within their building.

**Conclusion:**

We identified four potential strategies to increase attendance at CHAP sessions by residents of subsidized housing for older adults: strengthen confidentiality for those attending the sessions; use community peer networks to enhance recruitment; pair attendees to increase the likelihood of participation; and intervene through opinion leaders or bridging individuals.

**Supplementary Information:**

The online version contains supplementary material available at 10.1186/s12913-020-06019-2.

## Background

High blood pressure (hypertension) is one of the most important risk factors for death and disability in Canada and globally [1]. The Cardiovascular Health Awareness Program (CHAP) is a community-based, patient-centered hypertension prevention and management program targeting key modifiable risk factors and aimed at older Canadians. CHAP connects community resources with health system actors (primary health care providers, peer volunteers, local community organizations, and other health professionals) to improve participants’ cardiovascular health. During CHAP sessions, held in familiar settings such as pharmacies, places of worship, and other community spaces, participants’ blood pressure (BP) is measured following Hypertension Canada recommendations and their cardiovascular disease (CVD) risks are assessed. Trained volunteers help participants understand their risk profiles and provide information on locally available resources and support programs. With participants’ permission, BP readings and CVD risk profiles are shared with their physician [2, 3]. CHAP was previously successfully implemented and evaluated as a cluster-randomized controlled trial (cRCT) in 39 mid-sized communities in Ontario and was associated with a 9% reduction in annual hospital admissions for acute myocardial infarction, stroke, and congestive heart failure [2]. It has subsequently been adapted and implemented in multiple settings and for different populations, such as interdisciplinary primary care clinics, patients on a waitlist for a family physician, ethno-cultural minorities, and younger people [3–6].

Recently, CHAP was adapted to target older adults living in social housing buildings in Ontario and Quebec, and its effectiveness is being evaluated in a cRCT [3, 7, 8]. This adaptation of the CHAP program included, for the first time, group-based monthly educational sessions aimed at increasing cardiovascular health awareness to promote healthy habits and self-management among residents [3]. Older adults living in social housing represent a vulnerable population and are more likely to be affected by multiple chronic conditions such as CVD and diabetes [3], have more falls [9], and have poor health literacy [10]. Implementing a health promotion program targeting older adults in subsidized housing poses many challenges [7, 8, 11]. For instance, program success could be influenced by the dynamics within each building, depending on residents’ characteristics and turnover (deaths/departures) [7, 8]. Previous CHAP-like initiatives in subsidized housing showed differences in participation between buildings ranging from 14 to 52% [7]. Despite efforts and multiple follow-ups, high proportions of participants did not attend or were lost during the program [7].

Social network analysis (SNA) has proven effective when applied to support health program implementation or policy making by describing relational dynamics in networks of community organizations [12–16]. SNA shifts the object of study from individuals to their relationships with others [17]. Initially developed in the 1930s, SNA methods rapidly proliferated in many research fields, such as physics, epidemiology, public health, and medicine [17, 18]. Several studies have shown that the effects of an intervention can vary according to the beneficiaries’ social network dynamics [13, 19–23], which mediate whether people benefit from the program or not [12, 13, 22]. For example, programs implemented by community members identified as opinion leaders have been shown to be more effective [13, 24, 25] than programs offered by external organizations [14, 22, 26]. Identifying well-known and appreciated leaders from the community who influence opinion is crucial, as their support is critical to successful program implementation [12]. Because of their deep understanding of their community’s dynamics and needs [12–14], they can serve as key informants to help adapt the program to the local environment and thereby have an impact on its sustainability [12, 14]. Identifying the wrong person as a key agent to implement a program can interfere with the program’s benefits [12, 18]. Diffusion theory can help explain how new ideas and behaviors spread within communities and among groups [27, 28]. In the fields of health education and health behaviors, it has been applied to study how a new idea or practice penetrates [27] and spreads into a group [29] through interpersonal communication [28] and peer leaders [30]. SNA is thus ideally suited to evaluating the diffusion within a network of behaviors such as attending CHAP sessions [13, 17].

Despite its strong potential and obvious application to community-based programs, SNA is not sufficiently used in community-based program planning and evaluation, yet it has the potential to shed light on why people attend programs in their community or not [12–14]. The analysis of personal networks in relation to program participation is often neglected due to the difficulties of setting up SNA (time constraints and number of people to interview) [12–14]. SNA can generate a relational portrait of the environment [11] in which CHAP is implemented and help explain the effects of interpersonal interactions on differences in attendance across settings. Neighborhoods may play a very significant role for the older population [31–34]. Proximity and involuntary relationships (those not easily avoided) appear to represent the main types of interactions [31]. Such ties appear to generate conflicting relationships, particularly in or near poor neighborhoods in large urban areas [18, 31, 35]. Thus, rather than being a source of support, some relationships can have a negative impact on a person’s health [18, 35–38]. Positive and negative aspects of neighborhood relationships produce distinct effects, such as withdrawal and isolation or participation and feelings of belonging. Even when a relationship is a source of distress, it may sometimes have positive aspects that can be an important motivation to maintain it [34, 35, 38]. In this context, older adults will develop different proximity and distance strategies with neighbors [31, 34].

By identifying interpersonal dynamics among residents of buildings participating in the CHAP program, this study was intended to complement existing knowledge about the effects of social networks on the implementation of health promotion programs in the specific context of subsidized housing. Our aim was to better understand how such interpersonal dynamics can influence an individual’s decision to participate in health promotion activities, so that more effective recruitment strategies might be developed to enhance attendance. To this end, we used SNA to evaluate whether relationships and social structures affected residents’ participation in the program and could explain the between-building differences in rates of attendance at CHAP sessions. The specific objectives of this study were to: 1) describe the dynamics of relationships among residents in subsidized housing who attended CHAP sessions; and 2) examine the effects of these dynamics on program attendance rates.

## Methods

### Research design: social network analysis

We aimed to describe the social networks in two buildings, one with a low and one with a high CHAP attendance rate. SNA combines quantitative and qualitative research methods to study and measure relationships (friends, family, and neighbors) by analyzing different links—i.e., who is connected to whom—as objects of study [26, 39]. The network data we collected were composed of several measures used to identify whether there was a person or a group who had the power of influence, such as network opinion leaders or bridging individuals, i.e., individuals who are instrumental in reaching disconnected sub-groups [18, 40]. We also used the network data to identify the diffusion potential of an innovation (CHAP session attendance) in a network [41]. The qualitative data complemented the identification of leaders and clans, but also provided information on interpersonal behaviors.

### Setting

The implementation of CHAP in subsidized housing for persons aged 60 years and older was part of a cRCT in 28 buildings (14 intervention and 14 control) in Quebec (Montérégie) and Ontario (Niagara). The present SNA study was conducted in the Quebec arm of the RCT. The CHAP sessions took place once a month in community rooms of the buildings participating in the program, between September 2018 and June 2019.

The 3-h CHAP sessions, conducted by volunteers trained in assessment protocols, privacy/confidentiality, and consent, were supervised by a research nurse. Participants moved through a circuit of six tables/stations for BP measurement, anthropometric measures, and CANRISK and CVD risk assessment, all of which required 20–40 min. The remainder of the session consisted of group-based educational activities, in the form of workshops or conferences facilitated by different organizations or partners that addressed a different theme each month: diabetes, wellness, paramedic services, preparing for a visit to a family doctor, chronic pain, diet, physical activity, hypertension, and pharmacy services. Participation in the CHAP sessions was voluntary and on a free drop-in clinic basis. In collaboration with the residents’ association or a volunteer living in the building, all residents were invited via several recruitment strategies: meetings with social housing representatives or with residents’ associations, posters, flyers, door-knob flyers, and automated telephone reminders [3]. Participation was defined as attending at least one CHAP session. Rates of attendance in Quebec varied across buildings, from 23.5 to 49.1%.

### Data collection

The SNA data collection was conducted between April and May 2020, i.e., towards the end of the program’s implementation period. Two buildings were selected based on attendance rates (high and low) and similarity in overall settings (conditions, presence of a residents’ association, etc.). Given that the building with the highest participation rate (49.1%) also had ideal conditions according to the literature (green spaces, services, few residents per building [38]), we instead selected the building with the next-highest attendance rate (34.3%), whose setting was more comparable to the second (low-attendance) building. All residents of the two selected buildings were eligible to participate, for a total of 150 persons. We informed residents about the study through posters and our presence at CHAP sessions, and we recruited them directly at their door. Data collection was done at their apartment or in the community hall and lasted approximately 60 min per person.

The study involved two data collection phases, quantitative and qualitative, that were conducted concurrently. The quantitative phase consisted of gathering data to examine each person’s relationships to all others in their own building [40, 42]. This methodology involved interviewing at least 25% of all building residents, to ensure centrality measures as in-degree (well suited to identify opinion leaders [24]), group membership, and total degree (diffusion measure based on opinion leader diffusion model [25, 28]), as indicators of network position [43].

Network information was collected through sociometric questionnaires developed for this study, supplemented by coding grids indicating the names of residents identified by the respondent and the types of relationships. To assess the quality of these relationships, respondents were asked to designate each named person as a friend, acquaintance, neighbor, or ‘like family’. To identify opinion leaders and generate a portrait of the relational dynamics among residents, respondents were specifically asked, in relation to each named person, about shared activities, confidences, advice, or information, service exchanges, trust (full, moderate, none at all), conflicts, and other themes of potential interest [24, 25, 28, 37, 42, 44–46] (see Additional file 1).

Qualitative data were gathered on relationships, as well as on proximity and distance strategies between neighbors. For this second phase, we targeted our recruitment based on information from CHAP (sociodemographic and attendance profiles). Guided by the principle of content saturation [47], we aimed to interview five to seven persons from each of the following profiles: 1) attended three CHAP sessions or more; 2) attended one session only; 3) did not attend any sessions. The qualitative interviews were recorded and supplemented by coding grids (guide and grids developed for this study). Topics covered included everyday life (typical day), solitude/loneliness, and relationships with neighbors, family, friends, and acquaintances (support and conflicts). Because multiple factors can influence the establishment of ties with other residents, such as the presence of strong external ties (friendship, family) [31, 44], we included information on relationships outside the building in our analyses (see Additional files 2a and 2b).

### Analysis

For the quantitative phase, the data were analyzed with ORA (SNA software) using a structural analysis method [12, 13, 37]. This software measured clustering (group and sub-group membership), centrality (in-degree, total degree), and other significant network measures relevant to assess network dynamics. The qualitative phase was subjected to thematic analysis [47] performed with NVivo using the deductive/inductive method. Table 1 summarizes the measures used for the analysis [24, 25, 28, 40, 48, 49].

**Table 1:** Network measures used for analysis

Measures	Definition	Proposed analysis or use
In-degree centrality	Sum of links received by an actor (incoming links) or number of times (s) he is nominated by the members of the network divided by the total number of possible links.	A high percentage shows confidence and interest in this actor. Could help identify opinion leaders in each building.
Total degree centrality	Sum of incoming (named by others) and outgoing (named by others) links of an actor divided by the total number of possible links.	A high percentage indicates that an actor is more likely to be in the flow of information and thus retain a central diffusion position.
Group membership	Number of groups of which an actor is member.	This measure complemented the evaluation of an actor’s ability to diffuse information/behavior.

## Results

From the two buildings, 69 out of 150 residents (46%) participated in the study. Table 2 summarizes the respondents’ profiles.

**Table 2:** Identification of respondents (n%)

	Building 1 *n* = 42	Building 2 *n* = 27
**Respondent rate**	42%	54%
**Residents included in the network (named by respondents)**	75%	92%
**Men**	10	8
**Women**	32	19
**Data collection**		
Sociometrics	23	14
Interviews	19	13
**Average age**	73 years	70 years
Living alone	38	12
Couple	3	10
Widow	0	2
With another family member	1	3
**CHAP participation**		
Never	20	16
1	9	7
2 and more	13	4

### Maps of complete networks

Using the quantitative data to identify the people in their network and links with other known neighbors, even if they were not close to them [45, 50], we mapped the connections for 75% of Building 1 residents and 92% of Building 2 residents.

**Fig. 1:**
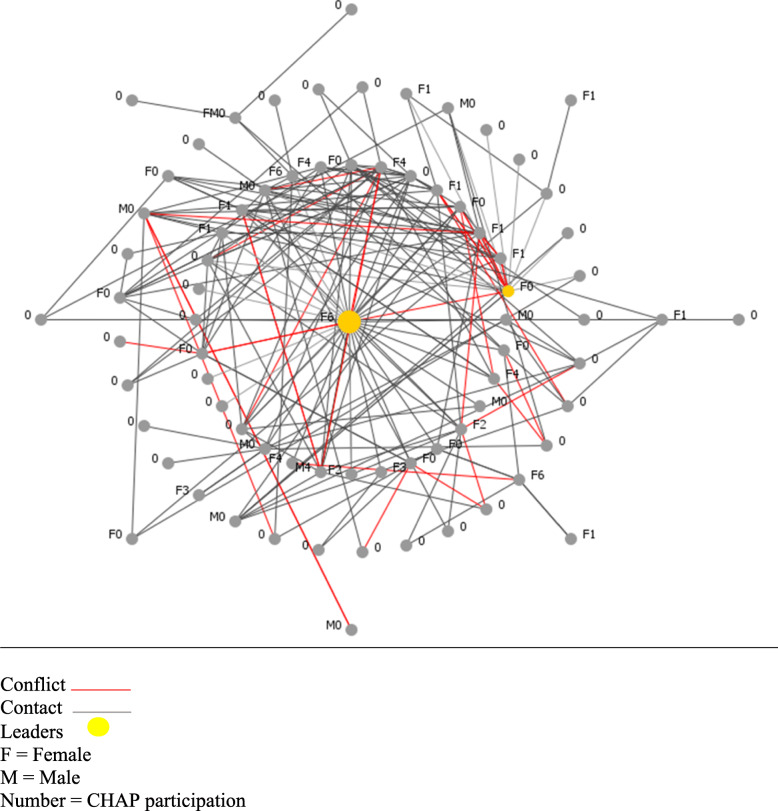
Complete network of Building 1 (links and conflicts). Conflict  .Contact . Leaders . F = Female. M = Male. Number = CHAP participation

**Fig. 2:**
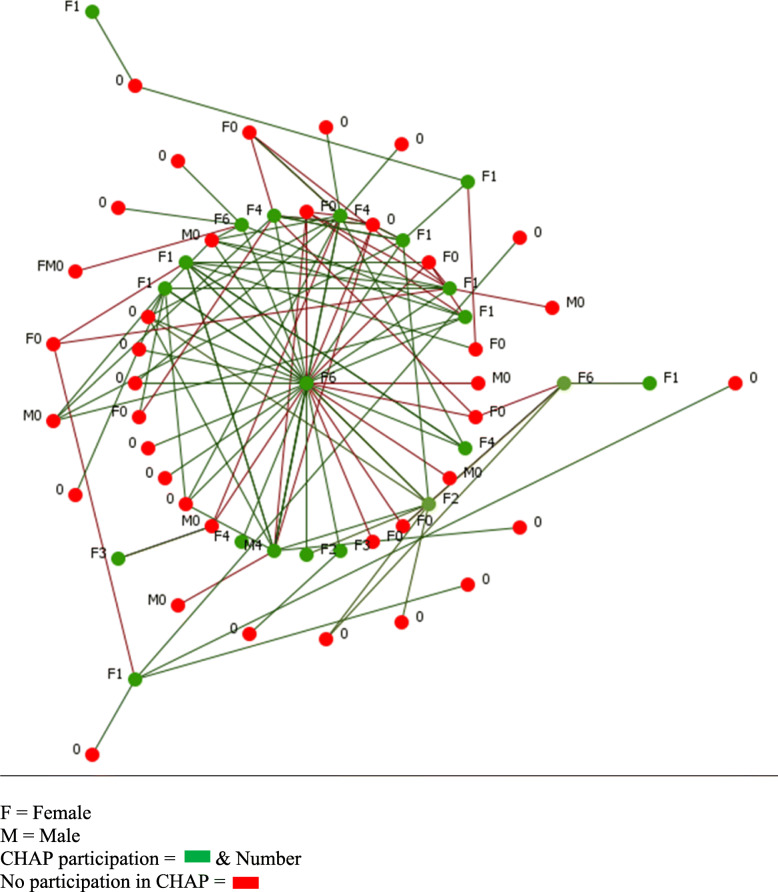
Diffusion potential of Building 1. F = Female. M = Male. CHAP participation =  & Number. No participation in CHAP =

**Fig. 3:**
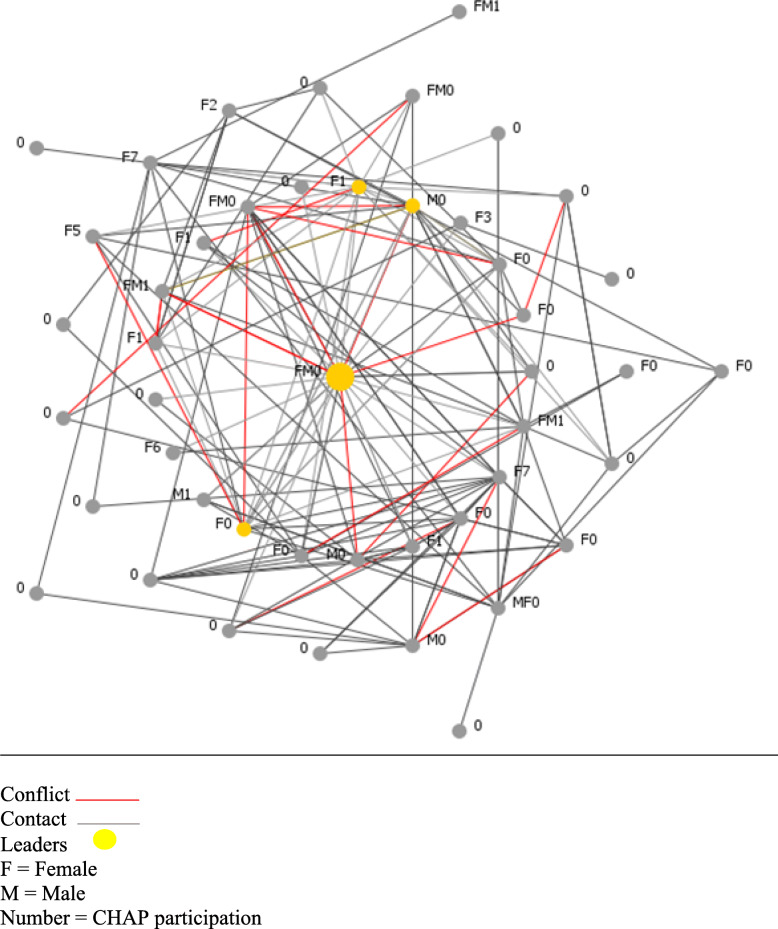
Complete network of Building 2 (links and conflicts). Conflict  .Contact . Leaders . F = Female. M = Male. Number = CHAP participation

**Fig. 4:**
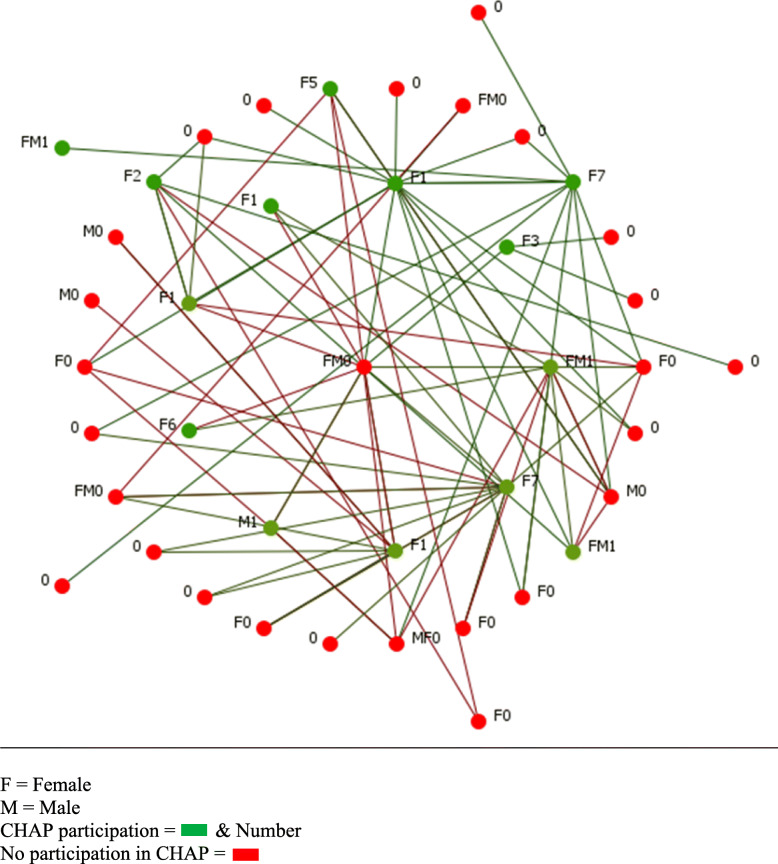
Diffusion potential of Building 2. F = Female. M = Male. CHAP participation =  & Number. No participation in CHAP =

The network maps of the two buildings (Figs. 1, 2, 3 and 4) present a type of visualization based on individuals’ positions in relation to each other, their identification of leading individuals, and the designations they attributed to other persons. Node names (0, M0, F0, etc.) represent gender (M = male, F = female) and number of CHAP sessions attended (0 to 7).

The maps in Figs. 1 and 3 illustrate the centrality of certain residents in the buildings and the distribution of conflicts. Persons in the center are those named most frequently by others in that building. Links in grey represent connections (knowing the neighbor), and links in red represent conflictual relationships with the named neighbor. Depending on the type of relationships residents identified from the coding grid, these are persons they consulted when they needed information or services, or wanted to attend building activities. Tables 3 and 4 summarize the networks data for each building.

**Table 3:** Networks data for Building 1

Nodes	75	
Edges	217	
In-degree	F6 23.2%	F0 9.1%
Total degree before	F6 18.2%	F0 11.6%
Total degree after	F6 28.4%	F0 0.17%

**Table 4:** Networks data for Building 2

Nodes	75			
Edges	217			
In-degree	FM0 32.7%	F0 20.4%	M0 14.3%	F1 2%
Total degree before	FM0 27.6%	F1 17.3%		
Total degree after	FM0 13.4%	F1 19.5%		

In the other two maps (Figs. 2 and 4), we removed the links representing conflictual relationships (red links), as well as the residents who did not attend CHAP and were not directly connected to someone who had attended (green nodes). These maps were intended to illustrate direct diffusion opportunities [25] between people in each building. In the Building 1 network configuration, we could reach 59% of residents and in Building 2, 82%. Removing conflict links allowed us to identify the main actors of diffusion in each building.

### Social networks in building 1: the leader makes the difference

In Building 1 (Fig. 1), which had a relatively high attendance rate (34.3%) at CHAP sessions, there were two clans (identified through qualitative interviews and network measures). A recent conflict between the leaders of these two clans had affected various activities, including CHAP session attendance. Figure 1 shows that one leader (F0) had many red links (conflicts), one of which was with the other leader (F6). Leader F0 did not attend any CHAP sessions because the other attended all sessions. F6 (in center) was a clan leader with 23.2% in-degree (‘named by others’ frequency). She attended all sessions, which may have contributed to this building’s high attendance rate. This leader position for F6 was confirmed after the conflict links were removed (Fig. 2), with a total degree (total of links when ‘someone named others’ and ‘is named by others’) of 28.4% for F6 and 0.17% for F0.

In addition, the in-degree ranking indicated that four out of six members of clan 1 attended at least one session, versus two out of six for clan 2 (Fig. 1), which illustrates the group influence of clan 1 on attendance. The map of diffusion potential in the Building 1 network configuration (Fig. 2) shows that the total degree remained high for leader F6, which means she remained a primary actor in diffusion and a key agent for the CHAP implementation (before: F6 = 18.2%, F0 = 11.6%; after removing conflict links: F6 = 28.4%, F0 = 0.17%).

### Social networks in building 2: the hidden bridge

In Building 2 (see Fig. 3), which had a lower attendance rate (23.9%), the leaders (a couple, FM0) had 32.7% in-degree and did not attend any sessions (0 out of 7). Again, there were two main clans in this building. The presence of conflicts indicated there were more conflicts around the central leaders than around the peripheral leaders. The in-degree ranking indicated that, of the six residents from clan 1, five did not attend at all and one attended once. In this network configuration (Fig. 4), we can see the total degree (in- and out-degree) changes between the leaders (FM0) and a CHAP participant (F1) who was not identified as central (only 2% in-degree). Before conflict links were removed, FM0 had 27.6% and F1, 17.3%. After conflict links were removed, F1 rose to 19.5% and FM0 declined to 13.4%, indicating that F1 was the most important person to promote program activities in this building. This person was, with M0 (in Fig. 3), a primary actor of diffusion and a key agent for the CHAP implementation, whom we should have involved in the program diffusion strategies. Thus, F1 was a common relation to the two cited main leaders and represented a kind of bridge in this divided network.

### Proximity and distance strategies

Qualitative analysis showed that residents of both buildings adopted proximity and distance strategies in their relationships with neighbors to various degrees. The first level of proximity strategies consisted of general polite behaviors: *“There’s this woman that I often chat with. She likes to sit on one of the chairs in the indoor lobby downstairs. Whenever I see her there, we wave to one another. You could say that this is a kind of interaction.... Yeah, we greet each other, and then maybe just say a few words, nothing more.” (R8/F1)*. Most people appreciated greetings and respect for an acceptable (low) noise level. Some were also willing to exchange services occasionally, which put them in closer relationships with some neighbors, especially those who were highly involved in the community and/or members of the residents’ association and local leaders. A greater level of proximity was also seen among those who had few [1, 2] people with whom they regularly exchanged services and visits: *“R: Sure, sometimes I’ll just go there or sometimes we just help each other out in small ways. For instance, if I make a big batch of homemade soup, I might bring some of it to neighbors who have done me a favor. These are the kind of encounters I have. Q: How about meeting for activities? R: No, that hasn’t happened.” (R3F/F0).* Proximity also took the form of shared activities and friendships: *“Well, I know [XY]: I see her pretty often, especially when there are activities down below, or whenever she walks her dog... and then there are [X and Y], who I would say are good friends.” (R2F/F0)*. They also demonstrated their connection by helping out in situations of illness or providing more substantial services during more serious illness, rather than only helping in emergencies. The highest level of proximity was to contribute to the community through volunteering or by serving on the residents’ association.

Residents participated in different activities for a variety of reasons, including socializing and forming new friendships. However, some did not need to participate in activities to rub shoulders with people. Some tended to become more involved because of others’ participation in the same activity: *“Yes, I did go to the CHAP session once. It’s the kind of thing I go to with [X] and his girlfriend. I had asked them to come with me …*. *because I’m not really all that comfortable going there alone yet. Once I get a better sense of who’s there and how things are done, I’ll probably go on my own. But right now, I’m still just checking it out.” (R9/F3)*. Respondents preferred to focus on the activity rather than on interaction between neighbors: *“When I do go to one of those places, it’s usually because I like going there and I want to learn something.” (R6/F4).*

The majority of respondents also mentioned using distance strategies. Several reported that they only helped others in emergencies, such as a power outage or a fall. Sometimes they charged for their services. A widely used strategy was to avoid neighbors in common spaces. Sometimes they wanted to avoid a particular person with whom they had a conflict: *“I try not to bump into her whenever possible. For instance, I avoid opening my front door when I know she’s on the other side.” (R1/F1)*. Sometimes they adopted this behavior as a habit: *“I go when I think no one is there, but if I see someone, I immediately turn around.” (R2/F4).* This strategy seemed to be used particularly to avoid conflicts or being judged: *“I’m just afraid of making a bad impression on others... because I worry about that a lot* …. *So I’ve become really withdrawn as a result. I can’t stand conflict.” (R1/F1)*, or engaging in gossip: *“I’m just not the kind of person who wants to get close to neighbors. Gossip turns me off.” (R3F/F0)*; or even being caught in clans: *“Yes, there can be tribalistic behavior here. In fact, that’s the main reason why I decided to distance myself from people I had established relationships with.” (R7/M0)*.

Reasons for conflict included noise, divergent opinions, personal attacks, and gossip, among others. Tensions also were created by personal attributes of certain neighbors, such as negative behaviors or strong personalities, sometimes due to medical conditions. Some conflicts were current, while others had occurred in the past, causing break-ups and ending the mutual assistance that once existed, such that some respondents were reluctant to form new relationships with neighbors. These same reasons also explained the isolation of some residents. Many appreciated or were accustomed to solitude. Some also were visited by relatives who lived outside the building, which influenced their withdrawal from neighbors and helped mitigate their loneliness. However, not all respondents appreciated solitude, and some expressed distress regarding their relationships with neighbors: *“Maybe it’s because I’m not in a good head space right now … because, you know, I don’t think I’m a bad person and I certainly don’t mean anyone harm, but I don’t like how people treat me here*. … *they remind me that I’m worthless, or worse. And so that’s how I feel now: worthless.” (R10/F4).*

This portrait needs to be nuanced by the fact that it was not possible to classify respondents strictly into these two separate types of strategies. The interviews showed a deeper ambiguity. For example, a resident might have helped a neighbor intensively during a certain period or been friends with them, and afterwards avoided this person because the relationship had become time-consuming, or because a problem of trust or poor health had made the respondent unable (or unwilling) to continue. In some cases, respondents used distance or proximity strategies, or a mix of both, in their daily life depending on their mood, their health, or the people they encountered: *“On one hand, I need to keep to myself and I need to live alone, and on the other hand, I don’t want to be all alone all the time. I do need to see people sometimes, and then after I do, I need to be alone again to get back to my peace and quiet.” (R6/F4).*

### Reasons for attending or not attending CHAP sessions

Reasons for attending CHAP sessions were related to the program’s objectives, namely, knowing their health status, checking their BP, following up with their doctor, acquiring new knowledge through workshops and conferences, and wanting to take care of themselves: *“I go once in a while. The last time, I only went to attend the nutritionist’s talk on healthy eating. I found it really interesting.” (R2/F4)*. Some attended the sessions for the program’s benefits and because, even if they were not looking for social interaction, they met someone there with whom they were comfortable: *“I go to those sessions to get my blood pressure checked. There’s only one or two people that I chat with. Because, as I see it … most women show up in pairs, they’re always in pairs... There’s one person I talk to regularly and we always have a good connection. That’s been my experience. Q: Is that really the extent of your interactions? R: I have very, very few. I’m telling you … I’m not comfortable with social interaction.... I think I’ve put a wall around myself.” (R2/F4)*. Some respondents had attended the CHAP sessions because a neighbor invited them. For example, respondent R1, who was generally isolated from the neighbors, did come once: *“When [X] told me about it and said she was going, I said to her, ‘Alright, I’ll come with you.’”* However, she added, *“The only reason I don’t go is because I don’t want to see any of my neighbors.”*

While some respondents did not feel the need to attend CHAP sessions because they were already being followed by their doctor, the main reasons mentioned for not participating were related to respondents’ relationships with their neighbors: *“Since I already have a family doctor, I can consult with her over the phone whenever I need to. I think maybe I just want to avoid being around certain groups in this building.” (R5/F0)*. They also mentioned the sometimes charged atmosphere between residents during the CHAP sessions as a barrier to attendance: *“I try to go but I’m not sure I’ll keep going... even though going means that I can get my blood pressure taken. Q: Are you disappointed? R: Yes, because I want to listen to the talks; I want to hear the expert’s health tips and what she has to say.” (R2/F4)*. Also, respondents did not want neighbors to be aware of their health status when their blood pressure was being taken: *“Yeah, well you know some of us may feel self-conscious about attending those sessions. Q: In the sense that your neighbors might see you there? R: Yes, that’s what I mean.” (R7/M0).*

In general, the CHAP was considered to be no different from any other activity in the building. Some respondents did not participate in any activities, including CHAP, because they generally preferred to avoid conflicts and vindictive behaviors: *“Personally, I go often. And I’ve noticed that it’s usually the same ones who tend to participate, too. For instance, 90% of people who show up for bingo are regulars. Q: From one of those two gangs? Have you seen there any of the women that you describe as mean-spirited? R: I guess so. To tell you the truth, I’m not sure* …. *I went only once* [to bingo] *and I kept to myself; I kept my head down.” (R11/F0)*; and especially gossip: *“There’s this 53 year-old woman who stopped coming to the sessions. I told her, ‘I miss you. I liked seeing you there.’ She replied, ‘There’s too much gossiping and I just can’t deal with it.’ I think she’s right to say there are many people in this building who like to gossip.” (R8/F1).* Finally, another reason mentioned was the presence of cliques and clans: *“There are tribalistic groups that have coalesced in this building. They get together downstairs and they say slanderous things*. … *It’s so draining; I can’t wait to get back up to my place when I hear them get started.” (R5/F0).*

## Discussion

The main objective of this study was to evaluate whether relationships and social structures among residents affected participation in the CHAP program. Many other studies have shown the importance of having a peer leader to successfully implement a community-based program [13, 21, 30, 51]. Mapping existing network dynamics and residents’ positions and links (power structure) within the building is highly relevant to identify internal leaders who could support program implementation [12, 16, 23]. Respondents were able to clearly identify leaders (people who were the most central) in the community from their perspective, but using SNA to map the network configuration showed that leaders can also have a negative influence on the community. Interestingly, the peripheral leader in Building 2’s network was unaware of his position and did not know he could improve the program’s implementation by attending sessions. Another interesting aspect of the relational dynamics in this building was that a person (F1 in Fig. 2) not identified as central had a combined membership in 20 groups within the network, just behind the most central person, who had 27, but with numerous conflicts. We were also able to identify marginal individuals or groups who had not attended CHAP sessions but were connected to individuals who had (diffusion maps 3 and 4); such insight may be useful to adapt program implementation strategies accordingly [12]. In a network with many cliques, a person who is able to reach many others through memberships in numerous groups is highly valuable as an opinion leader [28].

While our qualitative results identified the same distance strategies demonstrated in other studies [31, 33, 44], our application of these findings to the barriers and reasons for not attending CHAP appears promising. Respondents tended to consider CHAP to be no different from any other activity held in the building. Despite the program’s potential benefits for their health, their conflictual relationships with leaders in their building and/or other residents constituted the most important reason for their non-attendance. Our results complement those of studies on social participation, defined as taking part in activities that allow interactions with other members of the community [38, 52], and demonstrate its close association with social support and networks [38].

Our aim was to identify promising strategies to increase the CHAP attendance rate. From our analysis, four recommendations emerged for future implementation. First, when setting up programs in environments where participants live side by side, strong emphasis must be placed on confidentiality. The CHAP program already had some measures in place—training volunteers in confidentiality issues, placing tables as far away from each other as possible, respecting participants’ privacy when measuring waist circumference, etc.—but given the conflictual relationships among residents, more should be done, especially when measuring BP. Second, respondents said in the interviews that they would go if they knew for sure that someone else they knew in the building was also planning to attend. Based on studies of other successful programs, one promising strategy might be to raise awareness among participants of their role as potential change agents and to use community peer networks to recruit and reach as many members of the community as possible [12, 14, 28, 29, 51]. Third, as the majority of respondents reported having a trusted person in the building, encouraging them to come in pairs could help to overcome barriers, such as fear of rejection by an already formed group. Fourth, in a context of conflict, if a power dynamic is identified in a building (clans competing for control of activities), intervening through the positive leader may be the most effective way to create beneficial change [25, 28]. On the other hand, in situations of conflict and/or negative leaders, intervening through bridging individuals might be more effective [18].

This study presents two main limitations, particularly in relation to changes that might occur in the network between the beginning and the end of the program implementation, as well as changes potentially induced by the presence of researchers for a portion of that time. A significant event, such as conflict between leaders or the death/departure of a leader, can considerably influence network dynamics and people’s participation in activities, including CHAP. Nevertheless, the advantage of conducting research in this type of micro-society setting is that we became aware of such events as they arose over the course of the study, because respondents reported them. However, given high resident turnover, changes in leadership in the buildings, and the emergence of conflicts that affect relational dynamics in general, it would be ideal if network data could be collected at several points over the four implementation phases: needs assessment, program design, implementation, and program sustainability [12]. The program’s potential for diffusion in the community can be estimated and longer-term implementation strategies can be considered [12, 50]. Also, replicating the research in different buildings to establish a referential typology of relational dynamics could help counter these limitations related to changing relational dynamics.

## Conclusion

The objective of this study was to generate a portrait of two buildings’ relational dynamics, by examining cases with different rates of attendance (high and low), to see whether relationships could explain these differences. We used an SNA methodology to understand each building’s relational dynamics and their influence on program participation. The quantitative measures of social networks helped identify positive and negative leaders, to inform and support the program implementation. The qualitative results illustrated how not only conflictual relationships, but also the proximity and distance strategies used by residents, can influence participation in health promotion programs in social housing. The analysis revealed the presence of clans and conflicts, CHAP’s amalgamation (in the minds of residents) with other activities in the building, and other individual and relational factors to explain the differences in participation. It also helped identify well-known and appreciated leaders, which could inform future CHAP implementation planning to enhance attendance.

## Supplementary Information

**Additional file 1.** Sociometric questionnaire - CHAP Rel.

**Additional file 2.** Qualitative interview guide - CHAP Rel.

**Additional file 3.** Quali coding grid - CHAP Rel.

## Data Availability

Anonymized data can be made available on demand (contact Nadia Deville-Stoetzel).

## Abbreviations

BP: Blood pressure
CHAP: Cardiovascular Health Awareness Program
cRCT: Cluster-randomized controlled trial
CVD: Cardiovascular disease
SNA: Social network analysis
