# Supplementary material for: Participation in the Cardiovascular Health Awareness Program (CHAP) by older adults residing in social housing in Quebec: Social network analysis
Source: BMC Health Serv Res. 2021 Jan 7;21:37. doi: 10.1186/s12913-020-06019-2 (PMC7791708; doi:10.1186/s12913-020-06019-2)
Supplement: Supplementary file 1 — Additional file 1. Sociometric questionnaire - CHAP Rel. [file 12913_2020_6019_MOESM1_ESM.docx]

**SOCIOMETRIC QUESTIONNAIRE**

**ON NEIGHBORHOOD RELATIONS AND PARTICIPATION IN THE CARDIOVASCULAR HEALTH PREVENTION AND AWARENESS PROGRAM**

Cardiovascular health awareness program: portrait and mobilization of residents' social networks to support the implementation of the program in subsidized housing

Nadia Deville-Stoetzel, Janusz Kaczorowski and Magali Girard

Crossroads for innovation and health assessment - Research
Center of the University Hospital of the University of Montreal (CRCHUM)

Interview number:  Interview date:

Last name, first name:

Apt #:

Year of birth:

**How long have you lived in the building? ___________________**

**We will now see with you the relationships you have with the other people in the building.**

- **With which people in the building do you Exchange or Can you exchange Services and what type (example of types of services) and / or exchange Goods and / or Money and / or Visits.**
- **In the building, who do you go to if you need advice? What kind of advice? Health, CVD, lifestyle, Personal, PRactical, Relational? When you interact with neighbors do you ever give advice? What kind of advice? Health, CVD, lifestyle, Personal, PRactical, Relational?**
- **With whom do you do activities? What kind of activity?**
- **How long have you known this person?**
- **How often do you see this person (occasionally, regularly, every day or almost)?**
- **How would you qualify the relationship you have with this person? Are they members of your Family, FRiends, Acquaintances, Neighbors?**
- **With whom have you had conflicts before? Experienced Bullying? Is the relationship arranged better, Worse, Equal?**
- **According to you, which person has a positive influence or negative? Which are isolated? Can you identify clans?**
- **Which people do you confide in (Yes) ? If you don't confide in anyone: if necessary, who could you confide in (IN)? In whom you will not confide in any case (No) !**
- **How would you rate the confidence you have in this person? 1 Fully confident, 2 moderately confident, 3 Not at all confident in this person**

**Relationships with other people in the building**

| **Names**  **(Apartment number)** | **Provides services**  **Possibility (P) of providing services**  **G, S, M, V** | **Get services from**  **Possibility (P) of obtaining services from**  **G, S, M, V** | **Receive Advice**  **Type**  **H / CVD /L / PE / PR / R** | **Give advice**  **Type**  **H / CVD /L / PE / PR / R** | **Activities**  **If yes Note the activity** | **Date** | **Freq.**  **O / R / D** | **Qualifies the relationship**  **F / Fr / A / N** | **Conflict**  **Bullyied**  **+ BWE** | **Leaders positive (L +), negative (L-), isolated (I) , clans (C1, C2 ...)** | **Confide**  **Yes**  **IN**  **No** | **Trust links**  **1 Fully**  **2 Mod**  **3 Not at all** | **CHAP**  $\boldsymbol{\surd}$ |
| --- | --- | --- | --- | --- | --- | --- | --- | --- | --- | --- | --- | --- | --- |
|  |  |  |  |  |  |  |  |  |  |  |  |  |  |
|  |  |  |  |  |  |  |  |  |  |  |  |  |  |
|  |  |  |  |  |  |  |  |  |  |  |  |  |  |
|  |  |  |  |  |  |  |  |  |  |  |  |  |  |
|  |  |  |  |  |  |  |  |  |  |  |  |  |  |
|  |  |  |  |  |  |  |  |  |  |  |  |  |  |
|  |  |  |  |  |  |  |  |  |  |  |  |  |  |
|  |  |  |  |  |  |  |  |  |  |  |  |  |  |
|  |  |  |  |  |  |  |  |  |  |  |  |  |  |

**We are now going to see with you the relationships you have with your network of friends, family and acquaintances outside the building (+ professionals from organizations / SSS workers) ? Can you give me names of people significant that are in your network***(significant person refers to people that often rubs and / or emotional connection. Note names and fill in the grid)*

- **How long have you known this person (if not family)?**
- **How often do you see this person (Occasionally, Regularly, every Day or almost)?**
- **How often do you communicate with this person by phone, internet, etc. (Occasionally, Regularly, every Day or almost)?**
- **How would you qualify the relationship you have with this person? Are they members of your Family, FRiends of childhood, (former?), COLLeagues of work, a (former?), EMPloyer , Acquaintances, VOlunteers of association, PROfessional (social workers, doctors ... etc)?**
- **Whom do you go to if you need advice? What kind of advice? When you interact with those around you, do you ever give advice? What kind of advice? Health, CVD, lifestyle, Personal, PRactical, Relational?**
- **With which people do you or Could you exchange Services and of what type (examples of services) and / or exchange Goods and / or Money and / or Visit.**
- **With whom do you do activities? What kind of activity?**
- **With whom have you had conflicts before? Experienced Bullying? Is this arranged relationship better, in Worse, Equal?**
- **Show the list of names and ask the person to tell you which people on the list he or she confides in (Yes)? If she's not confiding in anyone, ask: If needed, which people on the list could you confide in (IN)? Not everything? Who you will never confide in ! (No)**
- **How would you rate the confidence you have in this person? 1 Fully confident, 2 moderately confident, 3 Not at all confident in this person**

**Relationships with the network of friends, family and acquaintances out of the building**

| **Names** | **Date** | **Freq . meet**  **O / R / D** | **Fréq. com**  **O/R/D** | **Qualifies the relationship**  **F / Fr / A /**  **Coll / Emp /**  **Vo / Pro** | **Receive Advice**  **Type**  **H / CVD /**  **L / PE / PR / R** | **Give advice**  **Type**  **H / CVD /**  **L / PE / PR / R** | **Provides services**  **Possibility (P) of rendering services**  **G, S, M, V** | **Get from s services**  **Possibility (P) of obtaining services**  **G, S, M, V** | **Activities**  **If yes Note the activity** | **Conflict**  **Bullyied**  **+ BWE** | **Confied**  **Yes**  **IN**  **NE**  **No** | **Trust links**  **1 2 3** |
| --- | --- | --- | --- | --- | --- | --- | --- | --- | --- | --- | --- | --- |
|  |  |  |  |  |  |  |  |  |  |  |  |  |
|  |  |  |  |  |  |  |  |  |  |  |  |  |
|  |  |  |  |  |  |  |  |  |  |  |  |  |
|  |  |  |  |  |  |  |  |  |  |  |  |  |
|  |  |  |  |  |  |  |  |  |  |  |  |  |
|  |  |  |  |  |  |  |  |  |  |  |  |  |
|  |  |  |  |  |  |  |  |  |  |  |  |  |

**Survey**

**Section 1: Daily life & neighborhood**

1) How do you feel in your accommodation?

⬜ I feel very good in my accommodation

⬜ I feel moderately well in my accommodation

⬜ I do not feel well in my accommodation

*1a Do you feel safe in your accommodation?*

⬜ Yes, absolutely

⬜ Moderately

⬜ No, not at all

2) How do you feel in your building?

⬜ I feel very good in my building

⬜ I feel moderately good in my building

⬜ I do not feel well in my building

*2a Do you feel safe in your building?*

⬜ Yes, absolutely

⬜ Moderately

⬜ No, not at all

3) How do you feel in your neighborhood?

⬜ I feel very good in my neighborhood

⬜ I feel moderately well in my neighborhood

⬜ I do not feel well in my neighborhood

*3a Do you feel safe in your neighborhood?*

⬜ Yes, absolutely

⬜ Moderately

⬜ No, not at all

4) Are you satisfied with the proximity to shops?

⬜ I am very satisfied

⬜ I am moderately satisfied (shops are missing / too far away)

⬜ I am not satisfied (there is no or too little trade / too far)

5) Are you satisfied with the proximity of the activities?

⬜ I am very satisfied

⬜ I am moderately satisfied (there is not enough or too much activity / too far)

⬜ I am not satisfied

⬜ DNK

6) In general, how would you rate your satisfaction with your daily life?

⬜ I am very satisfied

⬜ I am moderately satisfied

⬜ I am not satisfied

**Section 2: Participation in the program**

7) Are you familiar with the Cardiovascular Health Awareness Program?

⬜ Yes

⬜ No

8) Have you ever attended a CHAP session in your building?

⬜ Yes (go to questions 8a, 8b and 9)

⬜ No (go to question 8b and 1 4 )

8a) **If yes**, which month did you start? __________________

8b) **If yes**, how often have you attended the CHAP sessions?

⬜ 1 time

⬜ 2 times

⬜ 3 times and more

              8 b) **If not**, for what reasons have you never participated in the CHAP? ____________________________________________________________________________________________________________________________________________________________________________________________________________________________ _________________________

**Questions for people who have already participated in the program:**

9) Do you still participate in the CHAP?

⬜ Yes (go to question 10 a)

⬜ No (go to question 10 b)

10 a) **If Yes**, for what reasons do you continue to attend PSSC sessions? *Several choices possible*

⬜ I have received information on my health

⬜ I am reassured about my state of health

⬜ I feel reassured of being followed and to receive information from the volunteers

⬜ I feel reassured to be followed by the nurse

⬜ To have my blood pressure

⬜ I find friends / acquaintances in the building

⬜ I can get out of my house a bit and socialize

⬜ Other (specify) _________________

10 b) **If No**, for what reasons did you stop attending the CHAP sessions? *Several choices possible*

⬜ I have not received relevant information (more than from my doctor or that I already know)

⬜ I feel more worried than reassured about my state of health

⬜ I do not feel reassured to be followed and to receive information from volunteers

⬜ I do not feel reassured to be followed by a nurse

⬜ I don't need to know my blood pressure

⬜ I don't get along with the people in the building who go there

⬜ I prefer to stay at home, quiet

⬜ Other (specify) _________________

10) How did you find out about the program?

*Several choices possible*

⬜ By posters in the building

⬜ By the door-knob flyers

⬜ By members of the tenants association

⬜ By friends in the building

⬜ Other (specify) _________________

11) In general, are you satisfied with the program?

⬜ Yes fully satisfied

⬜ Not entirely, some things bother me

⬜ No I am not satisfied at all

⬜ If not completely satisfied, specify for what reasons?

________________________________________________________________________________________________________________________________________________________________________________________________________________________________________________________________________

12) Do you ever discuss CHAP with other people? If so, with whom? (Check in the coding grid)

13) Have you created new relationships with residents of the building following the CHAP program?

⬜ Yes (circle names in coding list)

⬜ No

**Questions for everyone:**

14) What do you think can explain why some people do not participate in the program?

___________________________________________________________________________________________________________________________________________________________________________________________________________________________________________________________________________________________________________________________________________________________________

**SECTION 3: SOCIAL COHESION**

15) I feel like I am part of my community (neighborhood)

7

Strongly disagree

Disagree a little

Neither yes nor no

A little okay

Strongly agree

1

3

4

6

Disagree

Okay

5

2

 16) How do you rate your relationships with others in general?

*Several choices possible*

⬜ I participate in activities to create links

⬜ I participate in activities to be with others

⬜ I interact with others without doing any particular activity

⬜ I do an activity with others

⬜ I help others

⬜ I actively contribute to the community (eg volunteering)

17) How do you feel about living alone / Do you feel alone despite living as a couple? *Several choices possible*

⬜ I like being alone, I am a loner

⬜ I like solitude, but I also like the company of others

⬜ Loneliness weighs on me

⬜ DNK

18) Do you use social media (email, facebook, skype, etc)? *Several choices possible*

⬜ Yes to communicate

⬜ Yes to inform me

⬜ Yes to follow what is happening in their life (profile, photos, etc.)

⬜ No

**Section 4: Sociodemographic characteristics**

| **Gender** |  |
| --- | --- |
| Man | 🖵 |
| Woman | 🖵 |
|  |  |
|  |  |
| **Number of years in country / region? _________________**    **Country / region of origin : _________________**    **In general, would you say your health is**:    ⬜ Excellent  ⬜ Very good  ⬜ Good  ⬜ Fair  ⬜ Bad      **Family situation** |  |
|  |  |
| Single | 🖵 |
| Couple (no children) | 🖵 |
| Couple (have children) | 🖵 |
| Widower | 🖵 |
| With other family members | 🖵 |
| **Activity** |  |
| Employment | 🖵 |
| Volunteering | 🖵 |
| Other______________ | 🖵 |
|  |  |
| **Income**(more than one choice possible) |  |
|  |  |
| Employment income | 🖵 |
| Unemployment insurance | 🖵 |
| Income security (social welfare) | 🖵 |
| CSST | 🖵 |
| Retirement pension | 🖵 |
| Other______________ | 🖵 |
|  |  |
| **Profession:**________________ |  |
| **Highest level of education completed** |  |
|  |  |
| No certificate or diploma | 🖵 |
| High school diploma or equivalent | 🖵 |
| School certificate or professional diploma | 🖵 |
| College diploma | 🖵 |
| University certificate | 🖵 |
| Baccalaureate | 🖵 |
| Masters or doctorate | 🖵 |
|  |  |
| **Language spoken at home** |  |
|  |  |
| French | 🖵 |
| English | 🖵 |
| Other (specify) ____________ | 🖵 |
|  |  |
