# Supplementary material for: Participation in the Cardiovascular Health Awareness Program (CHAP) by older adults residing in social housing in Quebec: Social network analysis
Source: BMC Health Serv Res. 2021 Jan 7;21:37. doi: 10.1186/s12913-020-06019-2 (PMC7791708; doi:10.1186/s12913-020-06019-2)
Supplement: Supplementary file 2 — Additional file 2. Qualitative interview guide - CHAP Rel. [file 12913_2020_6019_MOESM2_ESM.docx]

**INTERVIEW GRID ON NEIGHBORHOOD RELATIONSHIPS AND PARTICIPATION IN THE CARDIOVASCULAR HEALTH PREVENTION AND AWARENESS PROGRAM**

Awareness Program cardiovascular health: portrait and mobilization of social networks residents to support the implementation of the program in subsidized housing

Nadia Deville-Stoetzel, Janusz Kaczorowski and Magali Girard

Crossroads for innovation and health assessment - Research
Center of the University Hospital of the University of Montreal (CRCHUM)

***General instructions****:*

*Whenever the respondent cites a person, must note the name in the coding grid and ask, at an appropriate time, the****questions of coding grid.***

*Pay attention****to the events****that the person will spontaneously address (diseases, accidents, financial loss, etc.). It is important to return to it in Section 2 to collect information on the help received at these times.*

**Section 1: Daily life**

Please tell me about your daily life? How is a typical day going? How do you feel on a daily basis?

⮚ *What are you doing? Where are you going? Whom do you see? Relation to solitude?*

Please tell me about where you live (apartment, neighborhood, city)? Activities and proximity to shops?

⮚ *Arrival in this accommodation, relationship with the house, accommodation*, *perception and relations with the neighborhood?*

Do you have relatives (children, siblings, etc.) or friends in the neighborhood?

⮚ If not, where do the people you see most often live? *(family members or friends : to be specified).*

If needed, could you find help from those around you? From whom, for example (friends, family, neighbors)?

We will now explore your everyday life based on events and the positions to see what types of assistance you receive or may receive.

*(We can ask questions around events such as)*:

- **Installation in your home***(furniture, financial assistance, help with moving)*: From whom did you find advice and support? What assistance have you received through your network? By whom and how? How did you go about furnishing your apartment? Did people from the building help you? Who? Do you know any people who lived here before moving in?
- **Fix things in the home**: What help have you received (or could you receive) through your network? By whom and how? Are people from the building helping you? Who?
- **Shop for groceries or go to appointments**Help received or can be received. Are people from the building helping you? Who?
- **In case of illness**or difficulty, who will you call in case of a health problem? For advice? Are people from the building helping you?
- **In case of money problem**Who? In the building?
- **In case of problem / need**Who do you confide in? Or if necessary, who could you confide in? In the building?
- **Other ideas.**

**Section 2: Network of significant people**

Tell me about the significant people in your daily life? Whether they are friends, neighbors or family (this can also involves community organizations)

*(Write the names in the coding grid and ask the following questions)*

- Since when do you know this person? How often do you see her (occasional, regular, every day or almost)?
- How would you qualify the relationship you have with this person? Who are these people? Are they members of your family, childhood friends, (former?) Co-workers, a (former?) Employer, acquaintances, association volunteers, professionals (social workers, doctors … Etc.)?
- How would you rate the confidence you have in this person?
- How do you assess, the relationship between the people who are part of your different circles (friends / family / neighbors, etc.)?

-------------

*(Show the list of names and ask the person to tell you)*

- With which person do you exchange services and what type of services (example of services) and / or exchange goods and / or exchange money.
- What activities do you do together?
- With which people have you experienced conflicts? For what reasons? Has the relationship improved or worsened with this person?
- With which person do you share information?
- Whom do you go to if you need advice and / or support needed? Or could you if needed?
- Whom do you confide in? Or if necessary, whom could you confide in? In the building?
- How does mutual support manifest in your family? With your friends? With your neighbors?
- During family celebrations with whom do you meet?

**Section 3:  Social cohesion and neighborhood relations**

What are your general impressions of your surroundings, relations between the people of your building *(if clans, ask the person to name the people in the clans)*, OMHL's social workers? How do you feel in the community (neighborhood)?

- Do you know the names of your neighbors? Do people greet you when they meet you on the stairs or other common area? Do you greet your neighbors when you meet them in common areas?

Do you sometimes receive or visit other people who live in the building or go out with them?

If you have support or a small favor to ask, are there people in your building that you could contact?

In general, what kind of relationship do you have with your immediate neighbors?

When you interact with neighbors, do you give advices (in healthy lifestyles, health, CVD)?

- To whom? How often (occasionally, often)?

According to you, who in the building has a good influence on others? a bad influence?

**Section 4: Social participation**

Do you know people who are isolated in the building? How many? And forwhat reasons, according to you?

Are you already involved in social, recreational, cultural (in connection with the culture of origin or that of friends), sport activities in the neighborhood in the past year? Activities in the building in the last year?

- How did you get the information about the activity? Did you make any contacts there? Do you continue to see these people?

Are there organizations, associations or groups in your neighborhood that offer services or activities that seem interesting to you?

- How did you get the information about these organizations? Did you already attend them? Did you make any contacts there? Do you continue to see these people?

How did you make contacts there (in general)?

**Section 5: Participation in the CHUM program**

Are you familiar with the Cardiovascular Health Awareness Program (CHAP)?

Have you ever attended a CHAP session in your building?

- If yes, How often? What are your reasons for continuing to participate in the program? Why did you stop participating in the program?
- If not, for what reasons do you not want to participate in the program? What would make you want to participate in the program?

**Questions for people who have already participated in the program:**

How did you find out about the program?

- If the person mentions another person, ask for details on their relationship with this person (see coding grid)

 What did you learn or take away from the CHAP sessions (lectures included)?

Do you ever talk about it with other people? If so, who? (see coding grid)

Are you satisfied with the program?

- Relationship with **volunteers**,
- General ambiance with other residents of the building

Have you created new relationships with building residents through the (CHAP) program? (If so, write down names and fill in the coding grid).

What do you think can explain why some people do not participate in the program?

How do you assess the way you participate in the activities in general (several choices possible)?

⬜ I participate in activities to create links

⬜ I participate in activities to be with others

⬜ I interact with others without doing any particular activity

⬜ I do an activity with others

⬜ I help others

⬜ I actively contribute to the community

Can you give me some examples?

**Section 6: General comments**

Do you have anything to add about neighborly relations and participation in the cardiovascular disease prevention program?

**Thank you for your precious collaboration!**

Interview number:  Interview date:

Last name, first name:

Apt #:

Year of birth:

**Section 7: Sociodemographic characteristics**

| **Gender** |  |
| --- | --- |
| Man | 🖵 |
| Woman | 🖵 |
|  |  |
|  |  |
| **Number of years in country / region? _________________**    **Country / region of origin : _________________**    **In general, would you say your health is**:    ⬜ Excellent  ⬜ Very good  ⬜ Good  ⬜ Fair  ⬜ Bad      **Family situation** |  |
|  |  |
| Single | 🖵 |
| Couple (no children) | 🖵 |
| Couple (have children) | 🖵 |
| Widower | 🖵 |
| With other family members | 🖵 |
| **Activity** |  |
| Employment | 🖵 |
| Volunteering | 🖵 |
| Other______________ | 🖵 |
|  |  |
| **Income**(more than one choice possible) |  |
|  |  |
| Employment income | 🖵 |
| Unemployment insurance | 🖵 |
| Income security (social welfare) | 🖵 |
| CSST | 🖵 |
| Retirement pension | 🖵 |
| Other______________ | 🖵 |
|  |  |
| **Profession:**________________ |  |
| **Highest level of education completed** |  |
|  |  |
| No certificate or diploma | 🖵 |
| High school diploma or equivalent | 🖵 |
| School certificate or professional diploma | 🖵 |
| College diploma | 🖵 |
| University certificate | 🖵 |
| Baccalaureate | 🖵 |
| Masters or doctorate | 🖵 |
|  |  |
| **Language spoken at home** |  |
|  |  |
| French | 🖵 |
| English | 🖵 |
| Other (specify) ____________ | 🖵 |
|  |  |
