# Supplementary material for: Participation in the Cardiovascular Health Awareness Program (CHAP) by older adults residing in social housing in Quebec: Social network analysis
Source: BMC Health Serv Res. 2021 Jan 7;21:37. doi: 10.1186/s12913-020-06019-2 (PMC7791708; doi:10.1186/s12913-020-06019-2)
Supplement: Supplementary file 3 — Additional file 3. Quali coding grid - CHAP Rel. [file 12913_2020_6019_MOESM3_ESM.docx]

**NEIGHBORHOOD RELATIONS AND PARTICIPATION IN THE CARDIOVASCULAR HEALTH PREVENTION AND AWARENESS PROGRAM**- Coding grid

*Each time the respondent quotes a person, the name should be noted in the coding grid, asked at an appropriate time, the****questions below :***

- Since when do you know this person? How often do you see them (occasionally, regularly, every day or almost)?
- How would you qualify the relationship you have with this person? Who are these people? Are they members of your family, childhood friends, (former?) Co-workers, (former?) Employer, acquaintances, association volunteers, professionals (social workers, doctors … Etc.)?
- How would you rate the confidence you have in this person?

-------------

- With which person do you exchange services and what type of services (example of services) and / or exchange of goods and / or exchange of money.
- What activities do you do together?
- With which people have you experienced conflicts? For what reasons? Has the relationship improved or worsened with this person?
- With which person do you share information?
- Whom do you go to if you need advice and / or support? Or could you if needed?
- Whom do you confide in? Or if necessary, whom could you confide in? In the building?
- Specify positive and negative leaders and presence of clans
- Do you ever talk about it with other people? If so, with whom?

| Names  Apt # | Date | Contact frequency (Occ, Regular, every Day or almost) | Qualifies the relationship  F / Fr / A /  Coll / Emp /  Vo / Pro | Trust | Exchange of services  (specify) | Exchange of goods and / or money  (specify) | Activities | Conflicts | INFos, ADVices, CONFidences  (several choices possible) | Leaders +and - and Clans | CHAP |
| --- | --- | --- | --- | --- | --- | --- | --- | --- | --- | --- | --- |
|  |  |  |  |  |  |  |  |  |  |  |  |
|  |  |  |  |  |  |  |  |  |  |  |  |
|  |  |  |  |  |  |  |  |  |  |  |  |
|  |  |  |  |  |  |  |  |  |  |  |  |
|  |  |  |  |  |  |  |  |  |  |  |  |
|  |  |  |  |  |  |  |  |  |  |  |  |
|  |  |  |  |  |  |  |  |  |  |  |  |
|  |  |  |  |  |  |  |  |  |  |  |  |
|  |  |  |  |  |  |  |  |  |  |  |  |
|  |  |  |  |  |  |  |  |  |  |  |  |
|  |  |  |  |  |  |  |  |  |  |  |  |
|  |  |  |  |  |  |  |  |  |  |  |  |
